# Re-defining Non-tracking Solar Cell Efficiency Limits with Directional Spectral Filters

**DOI:** 10.1021/acsphotonics.4c02181

**Published:** 2025-03-17

**Authors:** Alan R. Bowman, Samuel D. Stranks, Giulia Tagliabue

**Affiliations:** † Laboratory of Nanoscience for Energy Technologies (LNET), STI, École Polytechnique Fédérale de Lausanne (EPFL), Lausanne 1015, Switzerland; ‡ Cavendish Laboratory, Department of Physics, University of Cambridge, J. J. Thomas Avenue, Cambridge, CB3 OHE, United Kingdom; § Department of Chemical Engineering and Biotechnology, University of Cambridge, Philippa Fawcett Drive, Cambridge, CB3 0AS, United Kingdom

**Keywords:** solar cell, photovoltaics, energy, limiting efficiency, directional filter, halide perovskite, silicon, photonics

## Abstract

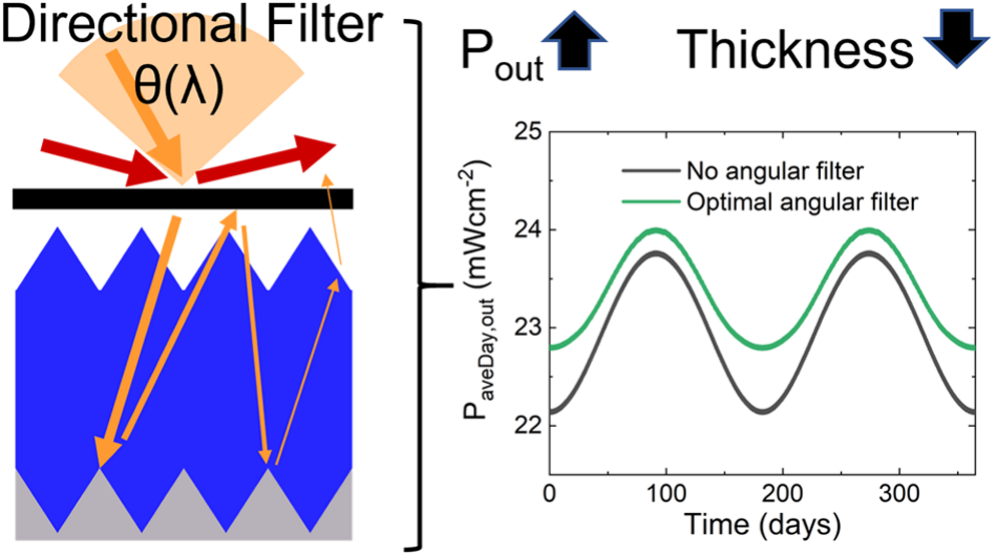

Optical filters that respond to the wavelength and direction of incident light can be used to increase the efficiency of tracking solar cells. However, as tracking solar cells are more expensive to install and maintain, it is likely that nontracking solar cells will remain the main product of the (terrestrial) solar cell industry. Here we demonstrate that directional spectral filters can also be used to increase the efficiency limit of nontracking solar cells at the equator beyond what is currently understood by up to ∼0.5% (relative ∼1.8%). We also reveal that such filters can be used to regulate the energy output of solar cells throughout a day or year, and can reduce the thickness of the absorber layer by up to 40%. We anticipate that similar gains would be seen at other latitudes. As this filter has complex wavelength-direction functionality, we present a proof-of-concept design based on Luneburg lenses, demonstrating these filters can be realized. Our results will enable solar cells with higher efficiency and more stable output while using less material.

## Introduction

Solar cells will become one of humanity’s main energy sources in the coming decades.^1^ Consequently, it is important to understand their efficiency limits so they can be fully optimized. The limiting efficiency of a single bandgap solar cell was derived by Shockley and Queisser^2^ and extended to real-world semiconductors by Tiedje et al., finding a 29.8%^3^ limit for silicon. Similar approaches have been applied to halide perovskite solar cells.^4^ The difference between the Shockley-Queisser approach and subsequent calculations is that, for real absorbers, a model of how the solar cell absorbs light is required. Typically Lambertian absorption is used, where the direction of light is randomized upon entering the solar cell.^5,6^ Introducing directional filters in front of a Lambertian absorber can increase solar cell absorption from specific directions, at the expense of lower light absorption from other directions. This concept has been applied to solar cells that track the sun,^7−9^ giving limiting efficiencies of 37% for silicon,^10^ and the realization of relevant filters has been discussed.^11−14^ To date the directional filters modeled have symmetric optical responses about their surface normal, which can be achieved via stacked dielectric layers. Filters with this symmetry constraint are detrimental for nontracking silicon solar cells with reasonable efficiencies (>10 μm thicknesses),^15^ as annual solar radiation is incident from a wide range of angles not symmetric about the solar cell’s surface normal, meaning these filters reduce total light absorption. However, there is no fundamental reason why directional filters need preserve symmetry about their surface normal, and the effects of removing this symmetry on nontracking solar cell efficiency has not been explored.

Here we present the effect of directional spectral filters with no symmetry constraints on the limiting efficiency of nontracking solar cells, exemplifying the concept on halide perovskites and silicon cells. Our study leads to three important conclusions: (i) the limiting efficiency of nontracking solar cells is higher than previously understood (relative increases of ∼1%/1.5% under AM1.5/at the equator, corresponding to an additional 17.5 kWhm^–2^ annually); (ii) it is possible to better regulate the annual energy output from nontracking solar cells; and (iii) directional filters can reduce material use (∼40% for silicon). Our results hold for nonideal solar cells and reduced direct solar irradiance, and we anticipate similar gains at other latitudes. Finally, as our study showcases the potential of directional spectral filters, we introduce a proof-of-concept filter design. This work presents a method for increasing nontracking solar cell efficiency, enhancing the deployment of solar energy.

## Main

**Figure 1:**
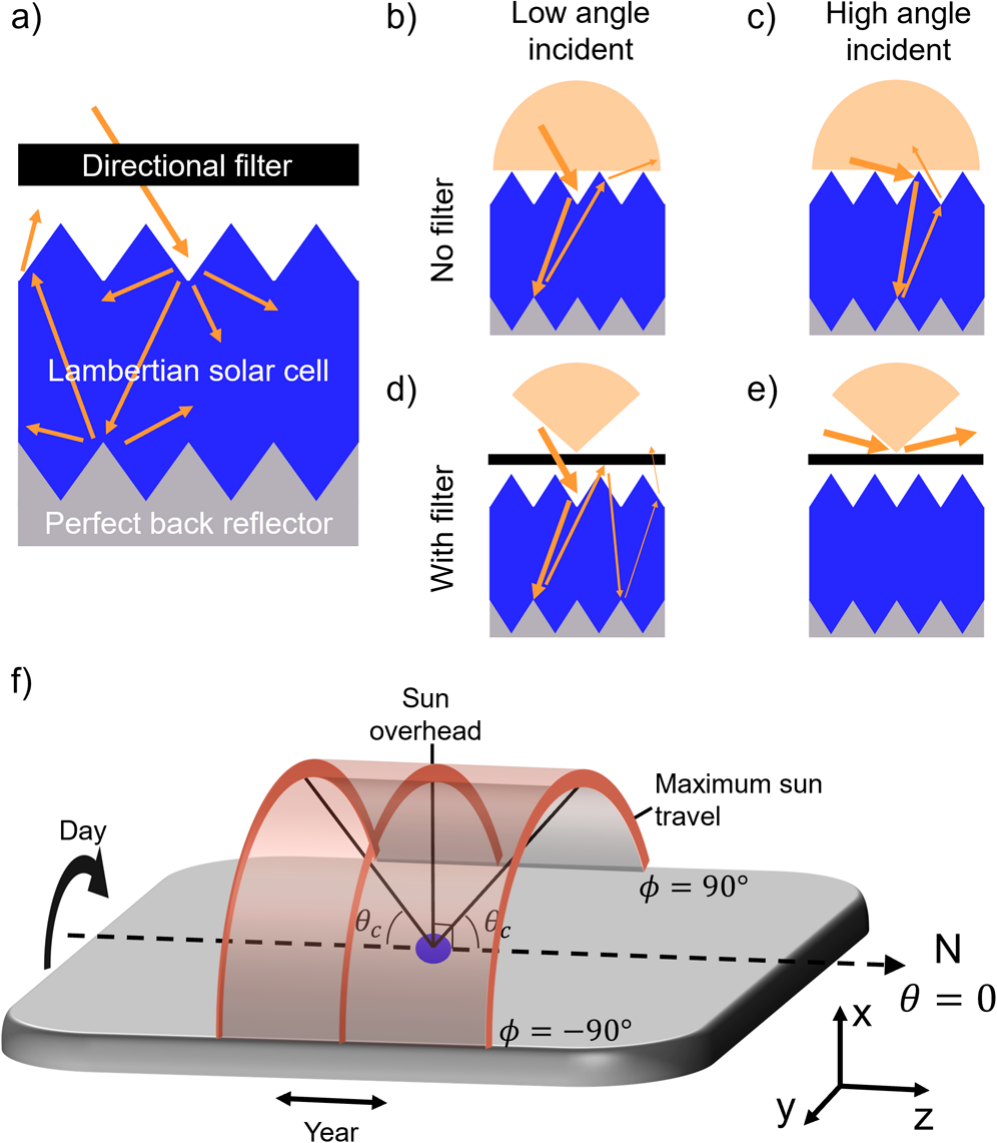
(a) Schematic of the idealized system modeled, consisting of a directional filter, a Lambertian absorbing solar cell and a perfect back reflector. (b, d) and (c, e) schematics of possible light paths for low and high incident angles of light (as marked in Figure) on a Lambertian absorber layer without/with a directional filter. (f) Schematic of how the sun moves throughout the year at the equator. Blue circle marks the location of the solar cell and N denotes north.

Solar cell efficiency calculations are based on detailed balance models^16^ that calculate the difference between absorbed and emitted radiation, and nonradiative losses, giving the current density out as a function of voltage *V*, i.e.
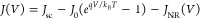
1Here, *J*_sc_ is the short circuit current density (describing absorbed radiation), *J*_0_ is the radiative recombination current density, *q* is the electronic charge, *k*_B_*T* is the thermal energy, and *J*_NR_ is the nonradiative loss current density. To calculate the limiting efficiency, all avoidable nonradiative loss processes are set to 0 and the power generated per unit area, *JV*, is maximized. Importantly for this work, *J*_sc_ and *J*_0_ are only functions of the solar cell’s absorption. Lambertian absorption typically describes these terms in limiting efficiency calculations: light’s direction is randomized when entering the solar cell and again upon reaching a perfect back reflector (Figure 1a^3^). Absorption for low and high incident angles of light is identical, due to this randomization effect (Figure 1b,c^5,6^).

We now consider placing a directional filter prior to the Lambertian absorber (Figure 1a). As we are studying limiting efficiencies, we do not discuss whether the filter is placed before or after an encapsulation layer, as this represents additional loss. Light incident within the filter’s accepted angular range impacts on the Lambertian absorber as normal. However, its escape cone is now reduced: while in Figure 1b light could exit the absorber at a high angle, it cannot with the directional filter present, increasing the optical path length and thus light absorbed in the absorber (Figure 1d). Conversely, light incident at high angles cannot enter the absorber (Figure 1e). Overall directional filters can be thought of as controlling an absorption budget: they increase absorption from certain angles, concurrently reducing absorption from other angles. However, the angle averaged absorption (the “budget”) remains fixed. This becomes mathematically absolute in the limit of weak absorption (Supporting Information, Note 1).

Directional filters increase the efficiency of solar cells that track the sun’s motion:^9^ as the majority of incident radiation is close to the panel’s surface normal, absorption from high angles can be reduced. Here directional filters have optically symmetric responses about the panel’s surface normal, with this functionality achieved through Bragg filters. Conversely, this filter design reduces efficiencies for nontracking solar cells^15^ as the sun is within the filter’s collection angle for a short period of time each day, and annual incident radiation is not symmetric about the panel’s surface normal.

To study the effect of directional filters with no symmetry constrains on nontracking solar cells we modeled devices at the equator with the cell’s and earth’s surface normals aligned. We developed a model of annual solar irradiance which includes both direct and diffuse irradiance but neglects clouds or other effects that reduce direct irradiance. We used this model of solar irradiation at the equator for two reasons: firstly, directional filters will provide the greatest efficiency increase when direct irradiance is largest relative to diffuse irradiance (i.e., at the equator), meaning our calculations represent the maximum possible efficiency increase from directional filters. Secondly, using real-world spectra would mean designing directional filters for specific points on the earth’s surface, while our aim is to identify general filter properties. We present how the sun moves throughout the year in our model in Figure 1f, with further details in Supporting Information Note 2: its polar angle oscillates sinusoidally throughout the year, while each day the sun rises in the East and sets in the West, following a linear change in azimuthal angle (see “day” and “year” arrows on Figure 1f). Our solar irradiances are based on AM1 solar flux calculated from the SMARTS program that was used to calculate AM1.5.^17−23^ A plot of direct and diffuse AM1 spectra is in Supporting Information, Note 3.

**Figure 2:**
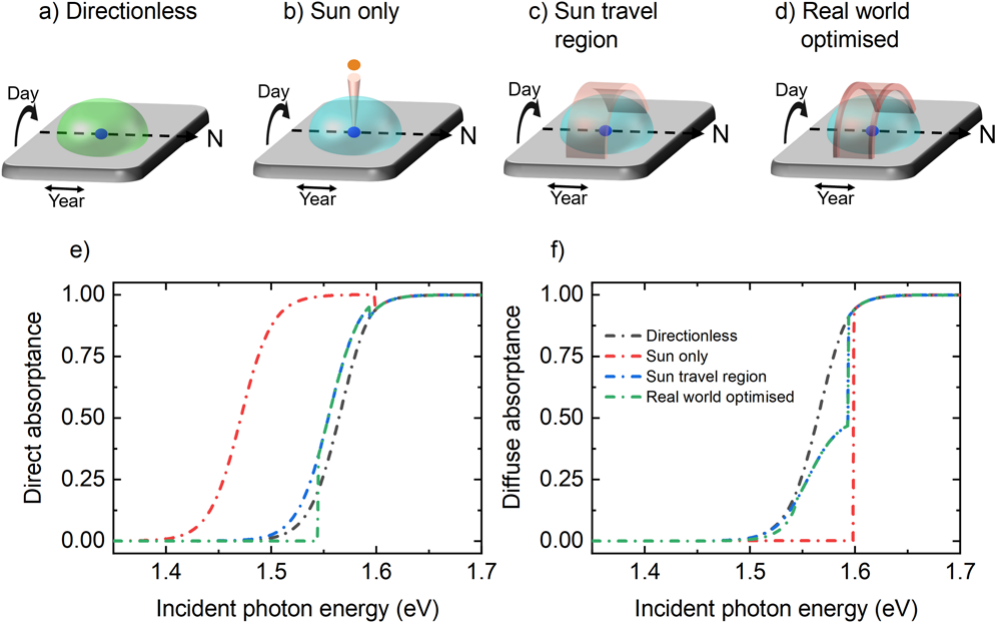
(a–d) The four absorption models considered in the main text. Specifically, “directionless” absorbs light equally from all directions, “sun only” absorbs light from only the angular region around the sun about the panel's surface normal close to its bandgap, “sun travel region” absorbs light from the region of the sky through which the sun travels at energies close to the material’s bandgap, and “real world optimized” has absorption directions to maximize light absorbed throughout the year at each wavelength, which is the same as “sun travel region” except at energies extremely close to the bandgap, which light is filtered to narrower polar angles. In (b), (c), and (d), the color of the directional filter approximately corresponds to the energy at which it should be applied, i.e., blue well above the bandgap, orange close to the bandgap and red extremely close to the bandgap. (e, f) Entire device direct and diffuse absorptances for incident photon energies for a 500 nm MAPbI_3_ film around its bandgap. Legend in (f) applies to (e) and (f).

We developed an approach to identify the optimal directional spectral filters for nontracking solar cells with no symmetry constrains, with details presented in Supporting Information, Note 3. Briefly, we derived *J*_sc_ for a Lambertian absorber coupled to a generic directional filter, and integrated this function over a year to obtain the light absorbed annually. This was evaluated for different filter designs coupled to real semiconductors (as the Lambertian absorber) through experimentally parametrized calculations. We modeled methylammonium lead iodide (MAPbI_3_) and silicon, using the same data as Pazos-Outón et al. for MAPbI_3_ and following our previous work for silicon.^4,24^ By employing an optimization algorithm (starting from random filter designs) we identified optimal directional spectral filters. We present results for MAPbI_3_ in the main text and equivalent results for silicon in Supporting Information, Note 4. To understand the effect of our filters, we compare our results to a Lambertian surface with no filter, termed *Directionless* (Figure 2a), and a filter that transmits light from the solar and circumsolar regions close to the bandgap, termed *Sun only* (Figure 2b), noting this design is optimal for a tracking solar cell. We find that at energies well above the material’s bandgap (∼0.1–0.2 eV), no directional filtering is best: here the materials modeled absorb strongly so directional filtering has no benefit. Conversely, near the material’s bandgap directional filters that only transmit light incident from the brightest regions of the sky are best. At the equator this corresponds to the region of the sky that the sun occupies throughout the year (between the θ_*c*_ angles marked in Figure 1f). This model, termed *Sun travel region* is presented in Figure 2c. Even stronger performance is obtained very close to the material’s bandgap by only accepting light close north-most and south-most positions of the sun, which have the maximum annual incident solar flux. This model, termed *Real world optimized*, is shown in Figure 2d and is discussed further in Supporting Information, Note 3. We highlight two additional points from these simulations: firstly, angular filtering in the azimuthal direction (as defined in Figure 1f) has no benefit at any energy. Secondly, for a solar cell at the equator absorption about the panel's normal should not be optimized, as this is not where the most solar energy is incident from (see Figure 2d and discussion below).

Direct and diffuse absorptances (i.e., absorption of light incident perpendicular to the panel and angle averaged) of all models are plotted in Figure 2e and f, respectively, for a 500 nm MAPbI_3_ thin film. *Sun only* has strong direct absorptance to much lower energies as only a small region of the sky is interacted with (substantially increasing the optical path length when compared to *directionless*). *Sun travel region* follows the same pattern to a lesser extent while *real world optimized* has three forms: at high energies directionless absorption is followed, at moderate energies *sun travel region* is followed, while at low energies direct absorption is 0, as absorption is focused in non-perpendicular directions. Importantly, all models have higher direct and lower diffuse absorption than *directionless*, implying higher solar cell efficiency. We also note that all absorptances except *directionless* contain step functions – this is not unreasonable in limiting efficiency calculations, as is discussed below.

We carried out efficiency calculations to quantify the effect of our filters. The limiting efficiency of each filter for a 500 nm MAPbI_3_ thin film under AM1.5 (i.e., laboratory test conditions) is presented in Table 1. *Sun only* gives the highest efficiency (35.3%) as it is designed to absorb light incident normal to the panel's surface, while *sun travel region* and *real world optimized* also give efficiencies higher than *directionless*. These results are discussed further in Supporting Information, Note 5, where we show that these filters: increase efficiency for different absorber thicknesses; increase both short circuit current and open circuit voltage; and give comparable efficiency increases when charge trapping rates are nonzero, i.e., these filters are beneficial for any solar cell.

**Table 1:** Efficiencies for 500 nm MAPbI_3_ Solar Cells with No Charge Trapping under AM1.5 and over a Year Placed at the Equator

absorptance model	AM1.5 efficiency (%)	equatorial efficiency over 1 year (%)
directionless	30.88	32.00
sun only	35.30	31.59
sun travel region	31.33	32.42
real world optimized	31.20	32.56

**Figure 3:**
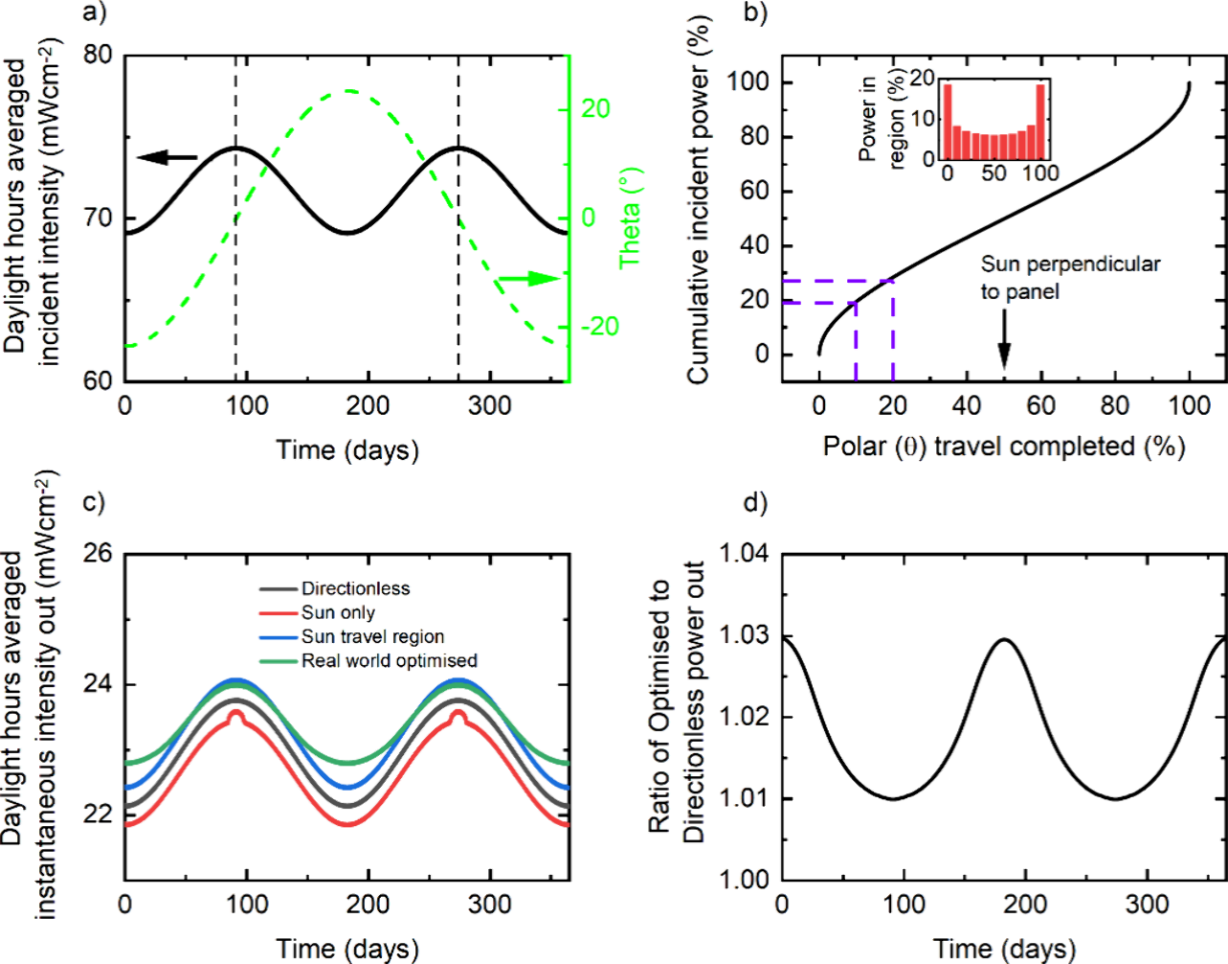
(a) Daylight averaged incident power with time (left-hand axis) and change in polar angle with time (right-hand axis, with 0° corresponding to the sun directly overhead, as marked by dashed vertical lines). (b) Cumulative percentage power incident as the sun travels across the sky. The inset in (b) splits the proportion of incident power out into different θ regions. (c) Daylight hours averaged instantaneous power out from absorptance models. (d) Ratio of the real world optimized to directionless models with time. All calculations are for 500 nm MAPbI_3_ absorber layers.

We also present limiting efficiencies of a 500 nm MAPbI_3_ thin film at the equator throughout a year in Table 1. As *sun only* absorbs light from a small region of the sky near the material’s bandgap, its efficiency is lower in the real world. Conversely, *sun travel region* and *real world optimized* give higher efficiencies than *directionless*. The relative efficiency increase is ∼1.8%, corresponding to an additional 17.5 kWhm^–2^ over a year. More strikingly, *real world optimized* generates energy at different times of the year. While the instantaneous incident power on the panel is highest when the sun is directly overhead (θ = 0 in Figure 3a), the sun spends more time at its minimum and maximum θ values (i.e., furthest north and south), meaning the total incident energy is highest from these angles. This is demonstrated in Figure 3b, where the cumulative incident power with θ shows more power incident from higher angles. We present the daylight hours averaged instantaneous intensity out throughout the year for all models in Figure 3c: *real world optimized* gives a more stable power output as it absorbs more light when the sun is at these extrema. More quantitatively, the ratio of *real world optimized* output to *directionless* output is presented in Figure 3d; at its extrema, up to 3% more solar energy is generated from *real world optimized*. Thus, directional filters can regulate output power throughout the year. We find comparable conclusions for silicon and that directional filters can reduce the optimal layer thickness from 74 to 46 μm (Supporting Information, Table S1), and we anticipate similar efficiency increases at other latitudes. Furthermore, in Supporting Information, Note 6 we explore reducing the proportion of annual direct solar irradiance to reveal that relative efficiency gains remain greater than 1% even when the direct portion of solar radiation is reduced by 70%. As expected, if all incident light is diffuse, then these filters do not result in any efficiency increase.

We now discuss the possibility of fabricating a filter to the design presented in Figure 2. The filter requires broadband response for all incident angles and need to be polarization independent. For a proof-of-concept design we propose combining two components: a layer that identifies the incident angle of light; followed by a layer that carries out spectral filtering. The first component can be a wide-field-of-view (WFOV) lens-type structure, focusing all incident angles to specific points at a surface, while the second structure a spatially varying collection of short-pass filters (i.e., filters that transmit/reflect light above/below a certain energy).

WFOV lenses are well-known in photography. However, photographic lenses are bulky and work with small entrance pupils.^25,26^ Conversely, here the ratio of entrance pupil to image size should be 1 to prevent energy loss. We used Monte Carlo ray tracing on simple lens structures to explore splitting different incident angles to different spatial positions (Supporting Information, Note 7). This revealed a trade-off between splitting angles and the fraction of transmitted light; whenever different angles were more strongly split, the fraction of light transmitted was lower. Thus, conventional lens structures are unlikely to achieve desired functionality.

WFOV metasurface lenses are a nascent technology that could increase the functionality and reduce the bulkiness of lenses. Successful demonstrations with quadratic phase profiles have proven effective for image reconstruction.^27−29^ However, they have low power transmission, especially for broadband light, and struggle to operate at incident angles higher than ∼70°. Therefore, while they may present a solution to realize required filter functionalities, with progress being made in that direction,^12^ we believe there is some way to go before demonstrating a metasurface ideal for this application.

**Figure 4:**
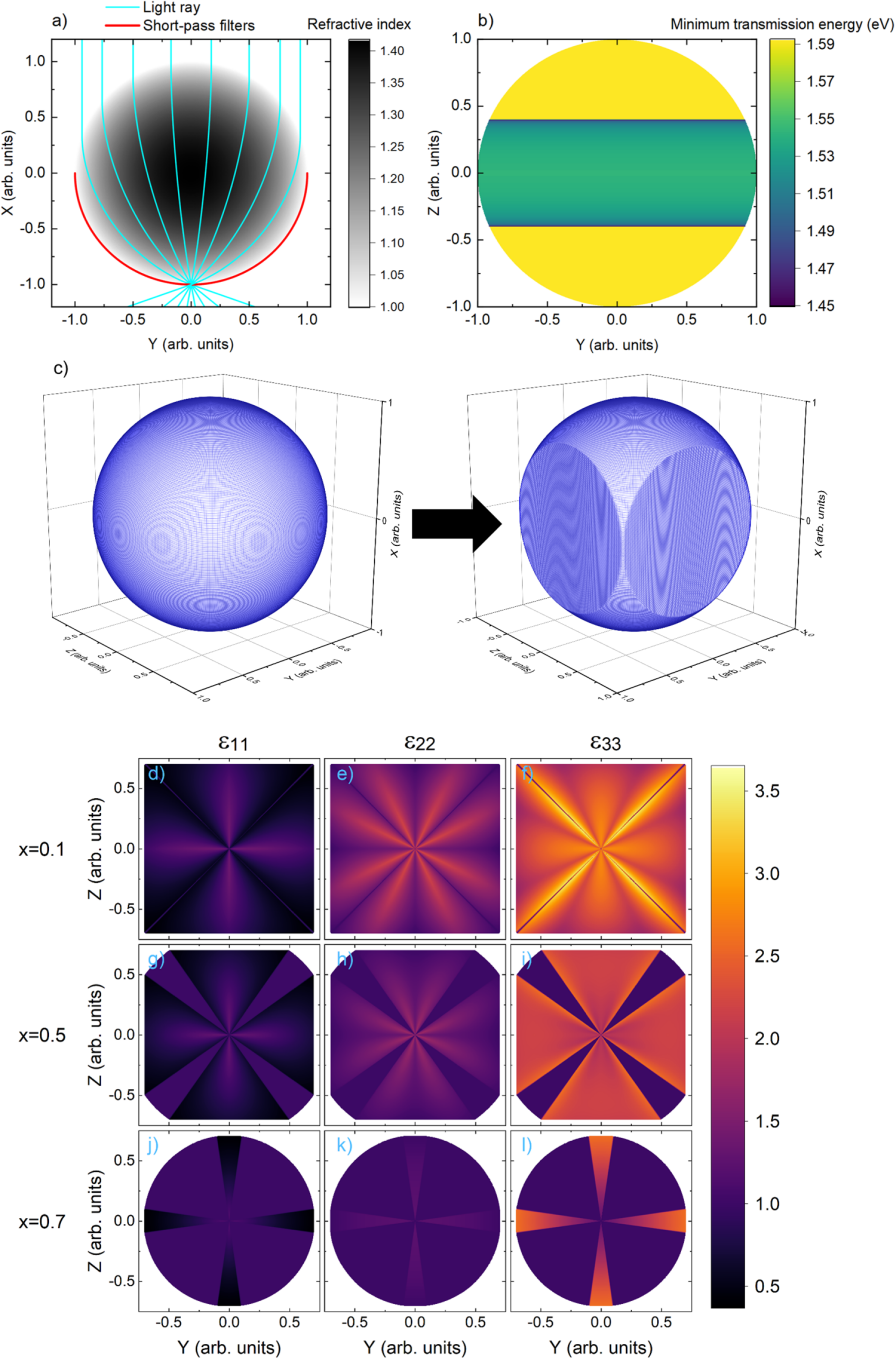
(a) Cross-section of Luneburg lens refractive index profile (z=0), with the effect on light rays passing through the lens shown in blue and the proposed location of the short-pass filters on bottom surface shown in red. (b) energy for short-pass filters on the bottom of the Luneburg lens to achieve “real world optimized” functionality. (c) Transform applied to Luneburg sphere to allow it to tesselate. (d–l) Relative permittivity (ε) along the three principal axes at three cross sections of the transformed Luneburg lens, as marked by x on the plots.

As this paper is on limiting efficiency calculations, we present an idealized filter with the required functionality and zero loss. This represents a proof-of-concept design rather than a filter suitable for real-world implementation. It shows that relevant filters can be achieved, with the design presented not realizable only due to current fabrication limitations. We base our design on a Luneburg lens coupled to ideal short-pass filters. A Luneburg lens is a spherical graded refractive index structure (i.e., with 0 loss) first introduced by Maxwell.^30,31^ They have been realized at radio and, via metasurfaces, near-infrared wavelengths.^32,33^ Luneburg lenses focus parallel rays of light incident on the structure to a point on the opposite surface (within the ray-optics approximation), as shown in blue lines on Figure 4a. This allows for the direction of incident waves to be identified. It now becomes straightforward to conceive of a structure that realizes *real world optimized* functionality – idealized short-pass filters placed on the bottom of the Luneburg lens (red line, Figure 4a). By varying the short-pass cutoff energy at each position on the lens, the desired functionality is realized. We present the short-pass filter energy cutoff as a function of position in Figure 4b. While challenging, we know of no intrinsic reason why short-pass filters with sharp steps and 100% transmission cannot be achieved, noting commercial filters are close to this. Thus, there is no fundamental reason why the step functions in Figure 2e,f are not achievable.

Our Luneburg lens-based design achieves the functionality of the *real world optimized* filter. However, it is spherical and cannot be tessellated, preventing it from being applied across a wide area of nontracking solar cell. Therefore, we employ transformation optics;^34^ starting from our Luneburg lens design, we produce a structure with the same functionality that can be tessellated, with the transformation considered presented in Figure 4c (see Supporting Information, Note 8 for details). Similar approaches have designed Luneburg lenses with flat bottoms and shown how to realize such devices with realistic materials.^35,36^ Transformation optics results in structures where electric and magnetic permittivities are not equal along the three principal axes. We present relative electric permittivity (assumed equal to relative magnetic permeability, following previous analyses^35^) along the three principal axes of the material at three cross sections of the new structure in Figure 4d–l. Close to the lens’ top surface (*x* = 0.7), the three permittivities are equal and approach 1, while further into the lens (*x* = 0.5, 0.1) they significantly vary from each other and contain discontinuities. Discontinuities can be fabricated, for example, by placing two materials adjacent to each other. Therefore, we have demonstrated a filter with no loss that can be realized, and that it can be tessellated to form a wide-area structure. Further work should focus on conceptually simpler filters, including accounting for interfacial losses, and explore filter designs at different points on the earth’s surface. Given the efficiency gains expected from these filters, they should increase the installation cost of solar cells by less than 1.8% to be economically viable. However, as solar cell modules represent less than 30% of solar cell installation costs, the filter can increase module costs by up to ∼6%.^37^

## Conclusion

We have demonstrated that directional spectral filters can increase nontracking solar cell efficiencies beyond what is currently understood, both in laboratory (AM1.5) tests and annually at the equator. We exemplify this through experimentally parametrized calculations on methylammonium lead iodide and silicon solar cells, with absolute efficiency increases of ∼0.5% (relative ∼1.8%) achieved. These filters also allow for less material to be used in the solar cell absorber and enable power output to be better regulated annually. Similar gains can be achieved for nonidealized solar cells and greater proportions of diffuse irradiation, and we anticipate similar gains at other latitudes. Finally, we present a proof-of-concept filter design. Further work is required to explore optimal filters at different latitudes and realize commercially relevant filter designs. Our study raises the bar for what is achievable from nontracking solar cells, bolstering their development and deployment, and will galvanize research into directional filters.

## Data Availability

The data and codes underlying this manuscript are available at https://doi.org/10.5281/zenodo.15020700.

## Supporting Information Available

Note 1 provides a derivation of the absorption budget concept; Note 2 discusses the model of the sun moving through the sky; Note 3 explains how solar cells were modeled and the derivation of different directional filter models; Note 4 presents results equivalent to those in the main text for silicon solar cells; Note 5 discusses AM1.5 efficiency calculations in more detail; Note 6 presents the effects of directional filters when the direct component of irradiance is reduced; Note 7 presents ray tracing calculations; Note 8 presents mathematics of the Luneburg lens transformation presented in the main text (PDF)
