# Supplementary material for: Re-defining Non-tracking Solar Cell Efficiency Limits with Directional Spectral Filters
Source: ACS Photonics. 2025 Mar 17;12(4):1739–45. doi: 10.1021/acsphotonics.4c02181 (PMC12007096; doi:10.1021/acsphotonics.4c02181)
Supplement: Supplementary file 1 — ph4c02181_si_001.pdf [file ph4c02181_si_001.pdf]

Supporting Information: Re-defining non-tracking solar cell efficiency limits with directional spectral filters

A. R. Bowman[1,2,3*], S. D. Stranks[2,3+] and G. Tagliabue[1+]

1. Laboratory of Nanoscience for Energy Technologies (LNET), STI, École Polytechnique Fédérale de Lausanne (EPFL), Lausanne 1015, Switzerland
2. Cavendish Laboratory, Department of Physics, University of Cambridge, J.J. Thomson Avenue, Cambridge, CB3 0HE, UK
3. Department of Chemical Engineering & Biotechnology, University of Cambridge, Philippa Fawcett Drive, Cambridge, CB3 0AS, UK

+corresponding authors: sds65@cam.ac.uk & giulia.tagliabue@epfl.ch

Number of pages: 22

Number of Figures: 12

Number of tables: 1

Brief comment on coordinate systems used in all Supporting Information Notes

We use two different angular coordinate definitions throughout these notes to aid with calculations. For angular coordinate systems without tilde (e.g. (θ, ϕ)), $\theta = 0$ is defined as the direction pointing North (noting the panel is at the equator) and ϕ takes values between $-\frac{\pi}{2}$ and $\frac{\pi}{2}$, corresponding to the region of the panel facing upwards (see main text Figure 1 for details). Conversely, coordinate systems with tilde (e.g. $(\tilde{\theta}, \tilde{\phi})$) have $\tilde{\theta} = 0$ defined as the direction perpendicular to the panel.

Supporting Information Note 1 – the concept of an absorption budget

Here we derive an absorption model for a Lambertian absorber with an arbitrary wavelength/directional filter above it, and demonstrate that in the case of weak absorption the concept of an absorption 'budget' emerges. The derivation of absorption follows a similar approach to that taken by Green[1].

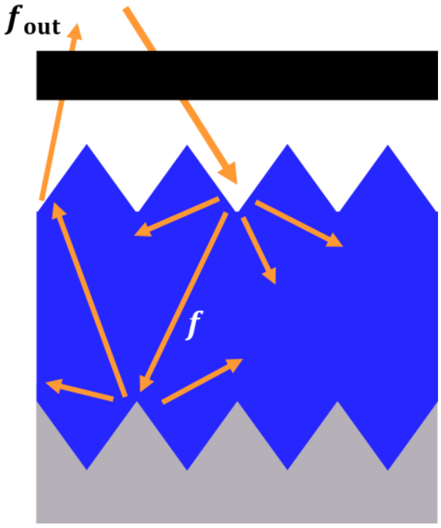

Figure S1. A schematic of Lambertian absorption.

A directional filter is placed above the solar cell and has a transmission as a function of angle defined as $g(\theta, \phi)$, where 1 corresponds to full transmission and 0 to full reflection. Here (θ, ϕ) are the polar and azimuthal angles defining the direction of incident light. We assume the solar cell to be a Lambertian absorber with ideal transmission at its front surface and full reflection at the back surface (as we are interested in limiting efficiencies). Thus, for light transmitted by the filter its direction is randomised upon contact with the absorber and fraction f is absorbed before it reaches the rear surface (Figure S1). Here the light's direction is again randomised and fraction f is again absorbed before it reaches the front surface. At the front surface fraction f_{out} escapes to the surroundings and the rest of the light repeats the randomisation and absorption process. This total light absorbed is given by a geometric sum of

$$g(\theta, \phi)(f + (1-f)f)(1 + (1-f)^2(1-f_{\text{out}}) + (1-f)^4(1-f_{\text{out}})^2 + \cdots).$$

Thus, we can write the absorbed light incident from a specific angle is

$$a_{\text{direct}}(\theta,\phi) = \frac{g(\theta,\phi) f(2-f)}{1-(1-f)^2(1-f_{\text{out}})}. \tag{S1}$$

Following Green[1], we can also define the fraction of light that escapes to the surroundings from the top surface of the absorber (noting that for non-absorbing structures, such as directional filters, $g(\theta,\phi)$ is the same for light incident from both sides of the filter):

$$f_{\text{out}} = \frac{\int g(\theta,\phi)\cos(\tilde{\theta}_{\text{in}})\, d\tilde{\Omega}_{\text{in}}}{\int \cos(\tilde{\theta}_{\text{in}})\, d\tilde{\Omega}_{\text{in}}}$$

where Ω denotes a solid angle in this Supplementary Note, the subscript *in* refers to angles inside the Lambertian absorber, which are defined through Snell's law as $n\sin(\tilde{\theta}_{\text{in}}) = \sin(\tilde{\theta})$, and the cos $(\tilde{\theta}_{\text{in}})$ factor arises from an area projection of the light's intensity (which is per unit area) onto the panel's area. Here n is the refractive index of the absorber. We note that $\cos(\tilde{\theta}_{\text{in}})\, d\tilde{\Omega}_{\text{in}} = \frac{\cos(\tilde{\theta}) d\tilde{\Omega}}{n^2}$ (as $d\tilde{\phi} = d\tilde{\phi}_{\text{in}}$)to give

$$f_{\text{out}} = \frac{\int g(\theta,\phi)\cos(\tilde{\theta}) d\tilde{\Omega}}{n^2\pi}.$$

In the limit of a weakly absorbing layer (i.e. $f \to 0$), $a_{\text{direct}}(\theta,\phi) \to \frac{2fg(\theta,\phi)}{f_{\text{out}}}$, meaning the light absorbed from a specific angle is given by

$$a_{\text{direct}}(\theta,\phi) = 2fn^2\pi \times \frac{g(\theta,\phi)}{\int g(\theta,\phi)\cos(\tilde{\theta}) d\tilde{\Omega}}.$$

Any quantity measuring the total absorption is of the form $\int a_{\text{direct}}(\theta,\phi)\cos(\tilde{\theta})\, d\tilde{\Omega} = 2fn^2\pi$. Therefore, while $a_{\text{direct}}(\theta,\phi)$ is a function of $g(\theta,\phi)$ and therefore depends on the directional filter, the total absorption is a fixed quantity and independent of the form of the directional filter. Thus, in the limit of weak absorption the total absorption is fixed and, to conserve this quantity, if absorption is increased in one direction it is correspondingly reduced in another: the absorption budget.

Supporting Information Note 2 – motion of the sun in the sky

We consider a model for how the sun moves through the sky, based on Figure S2.

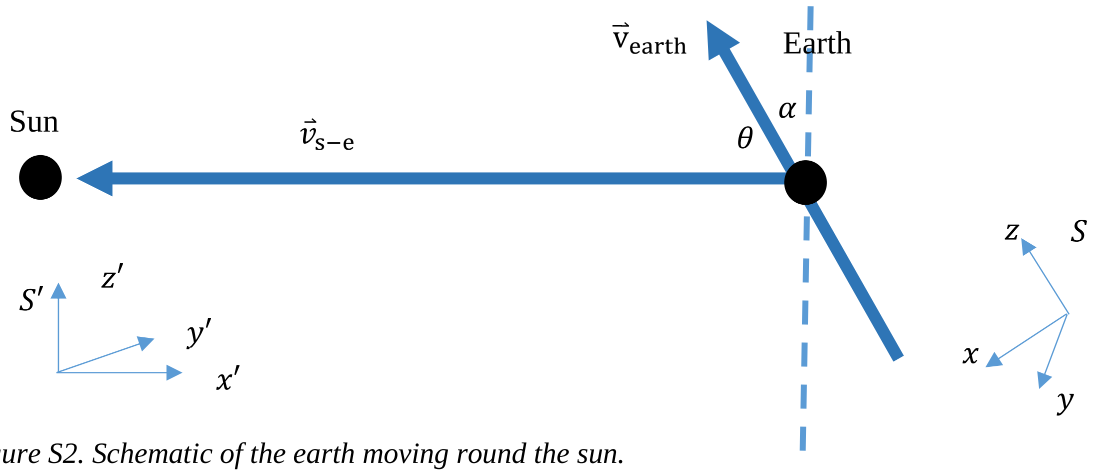

Figure S2. Schematic of the earth moving round the sun.

Over the course of a year we can say that, in the sun's frame S'

$$\vec{v}_{s-e}{}' = \begin{pmatrix} -\cos(\Omega t_{\text{year}}) \\ \sin(\Omega t_{\text{year}}) \\ 0 \end{pmatrix}$$

and the polar axis of the earth is defined as

$$\vec{v}_{earth}{}' = \begin{pmatrix} -\sin(\alpha) \\ 0 \\ \cos(\alpha) \end{pmatrix}.$$

Here $\Omega = \frac{2\pi}{T_{\text{year}}}$, T_{year} is the time for a year and $\alpha = 23.45°$. The angle between the axis of the earth and the sun at midday (θ) is given by

$$\cos(\theta) = -\vec{v}_{s-e}{}' . \vec{v}_{earth}{}' = \cos(\Omega t_{\text{year}})\sin(\alpha).$$

This equation describes how the sun's midday position polar coordinate, as defined in main text Figure 1f, oscillates for a solar panel placed at the equator. Furthermore, based on the definitions in Figure 1e, $\alpha = 90 - \theta_c$. For the azimuthal angle ϕ (as defined in Figure 1e in the rotating coordinate system S_{R}), we consider that each day the earth is fixed in a 2D plane (i.e. fixed θ) such that $\phi = \omega_{\text{day}} t_{\text{day}}$, where $\omega_{\text{day}} = \frac{2\pi}{T_{\text{day}}}$ and T_{day} is 24 hours. $T_{\text{year}} \approx 365 T_{\text{day}}$. In general our model considers the motion of the sun as follows: for a fixed θ, ϕ is varied through daylight hours. Then θ is incremented to the next day and the process repeated. This allows us to model the motion of the sun through the sky in a simple manner that captures its main features, rather than focusing on a specific year or starting condition.

We now derive a specific term useful to subsequent derivations. When the sun is directly overhead, in coordinate system S

$$\vec{\hat{v}}_{s-e} = \begin{pmatrix} \sin(\theta) \\ 0 \\ \cos(\theta) \end{pmatrix}.$$

The matrix S_{R} that describes the earth's rotation about its axis is

$$R_z = \begin{bmatrix} \cos(\phi) & \sin(\phi) & 0 \\ -\sin(\phi) & \cos(\phi) & 0 \\ 0 & 0 & 1 \end{bmatrix},$$

where we have chosen the direction or rotation to ensure the sun rises in the East. This allows us to write that in the earth's rotating frame

$$\vec{v}_{\mathrm{s-e,R}} = \begin{pmatrix} \sin(\theta)\cos(\phi) \\ -\sin(\theta)\sin(\phi) \\ \cos(\theta) \end{pmatrix}.$$

This vector defines the direction of the sun relative to the rotating earth using θ and ϕ as defined in main text Figure 1e. A key term in subsequent derivations is the cosine of the angle between an equatorial solar panel's surface normal and the sun. If the solar panel is at midday when $\phi = 0$, when we can write $\cos(\tilde{\theta}) = \vec{v}_{\mathrm{s-e,R}}.\vec{\hat{x}}_{\mathrm{R}} = \sin(\theta)\cos(\phi)$, where $\vec{\hat{x}}_{\mathrm{R}}$ is a unit vector along x in the rotating coordinate system (so parallel to the panel's surface normal at all times).

Supporting Information Note 3 – Absorptance models

1. *General absorption model*

The four absorptance models introduced in the main text, and how they are modelled, are outlined here. All models assume Lambertian-type absorption, a perfect back reflector and unitary front transmission. The start of this Note follows from Supporting Information Note 1, where we derived

$$a_{\mathrm{direct}}(\theta,\phi) = \frac{g(\theta,\phi) f(2-f)}{1-(1-f)^2(1-f_{\mathrm{out}})} = a_{\mathrm{direct}} g(\theta,\phi).$$

Here we have split $a_{\mathrm{direct}}(\theta,\phi)$ into an angle independent quantity, a_{direct}, and the angle dependent quantity $g(\theta,\phi)$ for simplicity in the following discussion.

Going beyond Supporting Information Note 1 and following Green[1], we can define the angle averaged absorption fraction f as

$$f = \frac{\int_{\tilde{\phi}_{\mathrm{in}}=0}^{2\pi} \int_{\tilde{\theta}_{\mathrm{in}}=0}^{\frac{\pi}{2}} \left(1-e^{-\frac{\alpha t}{\cos(\tilde{\theta}_{\mathrm{in}})}}\right) \cos(\tilde{\theta}_{\mathrm{in}}) \sin(\tilde{\theta}_{\mathrm{in}})\, d\tilde{\theta}_{\mathrm{in}}\, d\tilde{\phi}_{\mathrm{in}}}{\int_{\tilde{\phi}_{\mathrm{in}}=0}^{2\pi} \int_{\tilde{\theta}_{\mathrm{in}}=0}^{\frac{\pi}{2}} \cos(\tilde{\theta}_{\mathrm{in}}) \sin(\tilde{\theta}_{\mathrm{in}})\, d\tilde{\theta}_{\mathrm{in}}\, d\tilde{\phi}_{\mathrm{in}}} = 2\int_0^{\frac{\pi}{2}} \left(1-e^{-\frac{\alpha t}{\cos(\tilde{\theta}_{\mathrm{in}})}}\right) \cos(\tilde{\theta}_{\mathrm{in}}) \sin(\tilde{\theta}_{\mathrm{in}})\, d\tilde{\theta}_{\mathrm{in}}$$

where α is the material's absorption coefficient and t the absorber layer thickness. The tilde denotes a different coordinate system to that used in the rest of this Supplementary Note, with $\tilde{\theta} = 0$ parallel to the solar panel's surface normal, and the subscript *in* corresponds to angles inside the material (valid in the limit of weak absorption). We can also follow in Supplementary Note 1 to define

$$f_{\mathrm{out}} = \frac{\int\int g(\theta,\phi) \cos(\tilde{\theta}_{\mathrm{in}}) \sin(\tilde{\theta}_{\mathrm{in}})\, d\tilde{\theta}_{in} d\tilde{\phi}_{\mathrm{in}}}{\int_{\tilde{\phi}_{\mathrm{in}}=0}^{2\pi} \int_{\tilde{\theta}_{\mathrm{in}}=0}^{\frac{\pi}{2}} \cos(\tilde{\theta}_{\mathrm{in}}) \sin(\tilde{\theta}_{\mathrm{in}})\, d\tilde{\theta}_{\mathrm{in}} d\tilde{\phi}_{\mathrm{in}}} = \frac{\int_{\phi=-\frac{\pi}{2}}^{\frac{\pi}{2}} \int_{\theta=0}^{\pi} g(\theta,\phi) \sin^2(\theta) \cos(\phi)\, d\theta d\phi}{n^2\pi}.$$

Which follows from Snell's law, where n is the refractive index (taken to be the real part as we are working in the limit of weak absorption) and $\sin(\tilde{\theta})\, d\tilde{\theta} d\tilde{\phi} = \sin(\theta)\, d\theta d\phi$ to preserve solid angle elements. As discussed further below, we carried out a number of simulations to find the optimum values for $g(\theta,\phi)$. Our algorithms identified that, within our model, optimal values of g never had angular transmission going to 0 and then subsequently increasing to 1 again as $|\phi|$ is increased (noting the sun travels from $\phi = -\frac{\pi}{2}$ to $\phi = \frac{\pi}{2}$). This is justifiable from a physical point of view as most light in the sky always originates from when $\phi = 0$ and decreases in strength monotonically as $|\phi|$ is

increased. For ease of notation and explanation, we now present formulas within this approximation, with relevant full formulas presented below. We can now state that

$$f_{\text{out}} = \frac{2}{n^2\pi}\int_0^{\pi} \sin^2(\theta)\sin\big(\phi_l(\theta)\big)\,d\theta$$

where $\phi_l(\theta)$ contains all filter directionality information for the solar cell. Specifically, $g(\theta,\phi) = 1$ while $|\phi| < \phi_l(\theta)$ and is otherwise 0. At each θ value ϕ_l can take any value between 0 and $\frac{\pi}{2}$, with this value describing the maximum ϕ the solar cell can receive light from (at that polar angle). Note full symmetry in ϕ about $\phi = 0$ is assumed (as is the case for incident sunlight).

It is also necessary to define the diffuse (or angle averaged) absorptance, that is

$$a_{\text{diffuse}} = \frac{\int\int \ a_{\text{direct}} g(\theta,\phi)\cos(\tilde{\theta})\sin(\tilde{\theta})\,d\tilde{\theta}\,d\tilde{\phi}}{\int_{\tilde{\phi}=0}^{2\pi}\int_{\tilde{\theta}=0}^{\frac{\pi}{2}}\cos(\tilde{\theta})\sin(\tilde{\theta})\,d\tilde{\theta}\,d\tilde{\phi}} = n^2 a_{\text{direct}} f_{\text{out}}. \qquad (S2)$$

We note that in these absorption models the only term affected by the directional filter is f_{out}.

2. *Efficiency modelling details*

In all our modelling we split the incident light into two components – that which is incident directly from the sun, $\Phi_{\text{direct}}(E)$, assumed a point source which moves across the sky and is not always incident perpendicular to the panel, and diffuse light incident from the whole sky, $\Phi_{\text{diffuse}}(E)$. Here Φ is incident photon fluxes per unit area, per unit energy, per unit time. With these definitions the light absorbed at energy E, per unit energy, is

$$L(E) = a_{\text{direct}}(E)\Phi_{\text{direct}}(E)g(E,\theta,\phi)\sin(\theta)\cos(\phi) + a_{\text{diffuse}}(E)\Phi_{\text{diffuse}}(E)$$

while $J_{\text{sc}} = q\int L(E)dE$. Here $g(E,\theta,\phi)$ is now also a function of the photon energy and the $\sin(\theta)\cos(\phi)$ is the cosine between the direction of incident light and the perpendicular to the panel, derived in Supplementary Information Note 2. This arises from Φ_{direct} being per unit area, and this area needs to be projected onto the solar cell's area. We can also state that $J_0 = q\pi\int a_{\text{diffuse}}(E)\Phi_{BB}(E)dE$, where $\Phi_{\text{BB}}(E)$ is the black body flux per unit solid angle, per unit area, per unit energy, per unit time. Here θ and ϕ are functions of time, which defines how the current density changes throughout the day and year. This relationship is discussed in Supporting Note 2.

In all our calculations J_{NR} includes Auger recombination and, in the case of non-ideal solar cells, trapping as well. For the efficiency under AM1.5 we used the standard AM1.5 spectrum (split into direct and circumsolar for Φ_{direct}, and diffuse, for Φ_{diffuse}, contributions)[2]. For modelling the solar cell at the equator there is no generally available version of AM1. Thus, we generated the spectrum using the programme SMARTS, version 2.9.5. All inputs were kept identical to those used to produce the AM1.5 spectrum except the air mass was set to 1 and the angle of the solar cell to the surface tilt set to 0°. We note we used the Gueymard 2004 extraterrestrial spectrum (following example 6 of the software, which shows how to produce an updated AM1.5 spectrum)[3,4]. Optimal directional filter models were always calculated using AM1 spectra (even if the limiting efficiency was calculated for AM1.5). For further information on the software see[5]. All other solar cell modelling details are the same as our previous work[6]. We plot the AM1 spectrum in Figure S3, which demonstrates it has both direct and diffuse components. These are included in our model following equation S3 presented above. We note that $\Phi_{\mathrm{direct}}(E)$ includes direct and circumsolar components.

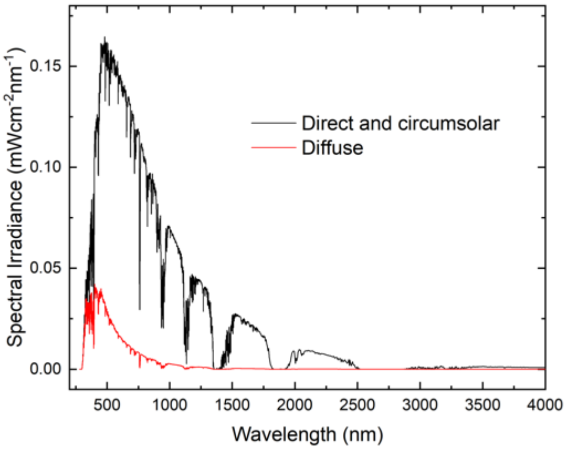

Figure S3. AM1 spectrum used in modelling.

3. *Absorption models*

   i. *Directionless*

Here $\phi_l = \frac{\pi}{2}$ for all θ values so $a_{\mathrm{direct}} = a_{\mathrm{diffuse}} = \frac{f(2-f)}{1-(1-f)^2\left(1-\frac{1}{n^2}\right)}$. We note this is the same form as previously derived by Green, which in the limit of weak absorption reduces to Yablonovitch's original formula[1,7].

   ii. *Sun only model*

The sun is a small disk in the sky, with angle from centre to edge $\tilde{\theta}_{\mathrm{sun}} = 2.5°$, giving $f_{\mathrm{out}} = \frac{\sin(\tilde{\theta}_{\mathrm{sun}})^2}{n^2}$. For a directional filter with symmetry about the normal to the solar cell that only accepts light from the solar disk, the absorption model is thus given by

$$a_{\text{direct}} = \frac{f(2-f)}{1-(1-f)^2\left(1-\frac{\sin(\tilde{\theta}_{\text{sun}})^2}{n^2}\right)}$$

and

$$a_{\text{diffuse}} = \frac{\sin(\tilde{\theta}_{\text{sun}})^2 f(2-f)}{1-(1-f)^2\left(1-\frac{\sin(\tilde{\theta}_{\text{sun}})^2}{n^2}\right)}.$$

To construct the *Sun only model*, at each energy we calculated the light absorbed when the sun is directly overhead as a function of energy ($L(E)$) for the absorption model just presented and the *directionless* model, and we select the model that gives the higher value (i.e. more perpendicular light absorption) – this corresponds to a wavelength and directional surface optimized for a tracking solar cell.

iii. Sun travel region and real world optimized models

Following Supporting Information Note 2 we model the sun moving across the sky from $\phi = -90°$ to $\phi = 90°$ at fixed θ i.e. the solar panel is placed at the equator with its normal parallel to the normal of the ground below it. Therefore, on a given day the solar and circumsolar light impacting on the surface of the solar panel is given by

$$I_1 = \Phi_{\text{direct}} \int_{-\phi_l}^{\phi_l} \sin(\theta)\cos(\phi)\, dt_{\text{day}}.$$

The $\sin(\theta)\cos(\phi)$ is the cosine of the direction perpendicular to the panel with the incident light, as at higher angles the projected area reduces (i.e. we neglect any atmospheric dispersion effects), see Supplementary Information Note 2 for further discussion. As $\omega_{\text{day}} dt_{\text{day}} = d\phi$,

$$I_1(\theta) = \frac{2\Phi_{\text{direct}}\sin(\theta)\sin(\phi_l(\theta))}{\omega_{\text{day}}}.$$

Now we consider vertical movement throughout the year. Approximating the sun as a point source we can state the light incident in half a year (noting that a full year is just 2 times this) is

$$I_2 = \frac{1}{T_{\text{Day}}} \int_0^{\frac{T_{\text{year}}}{2}} I_1(\theta) dT_{\text{year}}.$$

We already have the relationship between t_{year} and θ (see Supporting Information Note 2) so can write

$$I_2 = \int_0^{\frac{T_{\text{year}}}{2}} \frac{2\Phi_{\text{direct}} \sin\big(\phi_l(\theta)\big) \left(1 - \cos^2\big(\Omega t_{\text{year}}\big) \sin^2(\alpha)\right)^{0.5} dt_{\text{year}}}{2\pi}.$$

Finally, we make the substitution $u = \Omega t_{\text{year}}$ to obtain (noting $\Omega = \frac{2\pi}{T_{\text{year}}}$)

$$I_2 = \frac{\int_0^{\pi} 2\,\Phi_{\text{direct}} \sin\big(\phi_l(\theta)\big)\,(1 - \cos^2(u) \sin^2(\alpha))^{0.5} du}{2\pi\Omega}$$

This describes the total direct light that passes through the directional filter throughout the year. Multiplying by a_{direct} and including the diffuse light absorbed as well (integrated over the whole year) we can state the total light absorbed throughout the year is

$$G = \frac{2f(2-f)\Phi_{\text{direct}}}{\pi\Omega} \frac{\left(\int_0^{\frac{\pi}{2}} \sin\big(\phi_l(u)\big)\,(1 - \cos(u)^2 \sin(\alpha)^2)^{\frac{1}{2}} du + \frac{\pi\Phi_{\text{diffuse}}}{\Phi_{\text{direct}}} \int_0^{\frac{\pi}{2}} \sin\big(\phi_l(\theta)\big) \sin(\theta)^2\, d\theta\right)}{1 - (1-f)^2 \left(1 - \frac{4}{n^2\pi} \int_0^{\frac{\pi}{2}} \sin\big(\phi_l(\theta)\big) \sin(\theta)^2\, d\theta\right)}$$

Maximizing G by varying the form of ϕ_l is difficult as it is a function of functionals (with different integration limits for different functionals). We carried this out by using a stochastic optimization method: we assigned a random value of ϕ_l (between 0 and $\frac{\pi}{2}$) for each θ value. ϕ_l values were individually varied to increase G, and this was done iteratively until G was maximized. This was repeated for a wide range of random input parameters. We found G was always maximized when ϕ_l was equal to either 0 or $\frac{\pi}{2}$ at every θ value. This is still within the approximation of $g(\theta, \phi)$ being independent of ϕ, so we also derived the totally general form of G:

$$G = \frac{2\Phi_{\text{direct}} f(2-f)}{\pi\Omega} \frac{\left(\int_0^{\frac{\pi}{2}} \int_0^{\frac{\pi}{2}} g(u,\phi) \cos(\phi)\,(1 - \cos(u)^2 \sin(\alpha)^2)^{\frac{1}{2}} du d\phi + \frac{\pi\Phi_{\text{diffuse}}}{\Phi_{\text{direct}}} \int_0^{\frac{\pi}{2}} \int_0^{\frac{\pi}{2}} g(\theta,\phi) \sin(\theta)^2 \cos(\phi)\, d\theta d\phi\right)}{1 - (1-f)^2 \left(1 - \frac{4}{n^2\pi} \int_0^{\frac{\pi}{2}} \int_0^{\frac{\pi}{2}} g(\theta,\phi) \sin(\theta)^2 \cos(\phi)\, d\theta d\phi\right)}.$$

Again, we assigned a random value to $g(\theta, \phi)$ at every coordinate and varied these values to find a solution that maximises G, and we carried out this optimisation for a range of input absorption parameters. This confirmed that $g(\theta, \phi)$ is independent of ϕ and agreed with our 1-D (computationally faster) optimization of ϕ_l. We found that to maximise G there are three general solutions (we present these for the region $0 < \theta < \frac{\pi}{2}$, noting $\theta > \frac{\pi}{2}$ has the same results due to symmetry constraints), which we refer to as a), b) and c):

a) $\phi_l = \frac{\pi}{2}\ for\ all\ \theta$

This solution is identical to the *directionless* model, which gives

$$a_{\text{direct}} = a_{\text{diffuse}} = \frac{f(2-f)}{1-(1-f)^2\left(1-\frac{1}{n^2}\right)}.$$

Here

$$G = G_a = \frac{2k\Phi_{\text{direct}}\left(\cos(\alpha)\,E\left(\frac{\pi}{2}\middle|-\tan(\alpha)^2\right) + \frac{\pi^2\Phi_{\text{diffuse}}}{4\Phi_{\text{direct}}}\right)}{1-(1-f)^2\left(1-\frac{1}{n^2}\right)}$$

where $k = \frac{f(2-f)}{\pi\Omega}$ and $E(x|-\tan^2(\alpha)) = \int_0^x (1+\tan^2(\alpha)\sin^2(u))^{\frac{1}{2}}du$, which is a tabulated integral.

b) $\phi_l = \begin{cases} \frac{\pi}{2}\ for \frac{\pi}{2} \geq \theta > \frac{\pi}{2} - \alpha \\ 0\ elsewhere \end{cases}$

This solution gives

$$a_{\text{direct}} = \frac{f(2-f)}{1-(1-f)^2\left(1-\frac{2}{n^2\pi}\left(\alpha+\frac{1}{2}\sin(2\alpha)\right)\right)}$$

(noting the absorption is 0 when the direct light is incident from $0 < \theta < \frac{\pi}{2} - \alpha$) and

$$a_{\text{diffuse}} = \frac{2a_{\text{direct}}\left(\alpha+\frac{1}{2}\sin(2\alpha)\right)}{\pi}.$$

Here

$$G = G_b = \frac{2k\Phi_{\text{direct}}\left(\cos(\alpha)\,E\left(\frac{\pi}{2}\middle|-\tan(\alpha)^2\right) + \frac{\pi\Phi_{\text{diffuse}}}{2\Phi_{\text{direct}}}\left(\alpha+\frac{1}{2}\sin(2\alpha)\right)\right)}{1-(1-f)^2\left(1-\frac{2}{n^2\pi}\left(\alpha+\frac{1}{2}\sin(2\alpha)\right)\right)}.$$

c) $\phi_l = \begin{cases} 0\ for\ \theta < \frac{\pi}{2} - \alpha \\ \frac{\pi}{2}\ for \frac{\pi}{2} - \alpha < \theta < \theta_x \\ 0\ for \frac{\pi}{2} \geq \theta > \theta_x \ . \end{cases}$

Here we find

$$a_{\text{direct}} = \frac{f(2-f)}{1-(1-f)^2\left(1-\frac{2}{n^2\pi}\left(\theta_x + \alpha - \frac{\pi}{2} - \frac{1}{2}(\sin(2\theta_x) - \sin(2\alpha))\right)\right)}$$

(noting there is only absorption of direct light in the region $\frac{\pi}{2} - \alpha < \theta < \theta_x$) and

$$a_{\text{diffuse}} = \frac{2a_{\text{direct}}\left(\theta_x + \alpha - \frac{\pi}{2} - \frac{1}{2}(\sin(2\theta_x) - \sin(2\alpha))\right)}{\pi}.$$

Interestingly, in the limit of very small f, $a_{\text{direct}} \rightarrow 0.5$. For this solution,

$$G = G_c = \frac{2k\Phi_{\text{direct}}\left(\cos(\alpha)\, E(x|-\tan(\alpha)^2) + \frac{\pi\Phi_{\text{diffuse}}}{2\Phi_{\text{direct}}}\left(\theta_x + \alpha - \frac{\pi}{2} - \frac{1}{2}(\sin(2\theta_x) - \sin(2\alpha))\right)\right)}{1-(1-f)^2\left(1-\frac{2}{n^2\pi}\left(\theta_x + \alpha - \frac{\pi}{2} - \frac{1}{2}(\sin(2\theta_x) - \sin(2\alpha))\right)\right)}$$

where $\theta_x = \cos^{-1}(\cos(x)\sin(\alpha))$. Importantly, in this solution we still have the free parameter x which must be chosen to give the maximum value of G_c. This is given by the solution to

$$\frac{dG_c}{dx} = \tilde{G} = (1-(1-f)^2)\left(1 + \frac{\pi\Phi_{\text{diffuse}}}{\Phi_{\text{direct}}}\sin(\alpha)\sin(x)\right) + (1-f)^2\frac{2}{n^2\pi}\left(\cos^{-1}(\cos(x)\sin(\alpha)) + \alpha - \frac{\pi}{2} - \sin(cos^{-1}(cos(x)\, sin(\alpha)))\cos(x)\sin(\alpha) + \sin(2\alpha)\left(\frac{1}{2} - \sin(x)\, E(x|-\tan(\alpha)^2)\right)\right) = 0.$$

In general we solve this equation for x using a Newton-Raphson method, noting that the second derivative (required for this kind of solver) is

$$\frac{d\tilde{G}}{dx} = \sin(\alpha)\cos(x)\left(\frac{\pi\Phi_{\text{diffuse}}}{\Phi_{\text{direct}}}(1-(1-f)^2) - 2(1-f)^2\frac{2}{n^2\pi}cos(\alpha)E(x|-\tan(\alpha)^2)\right).$$

However, for small x (i.e. where absorption is extremely weak) an analytical solution can be derived:

$$x = \frac{(1-(1-f)^2)\left(\frac{\pi\Phi_{\text{diffuse}}}{\Phi_{\text{direct}}} + \left(\left(\frac{\pi\Phi_{\text{diffuse}}}{\Phi_{\text{direct}}}\right)^2 + \frac{(1-f)^2\frac{8}{n^2\pi}}{\tan(\alpha)(1-(1-f)^2)}\right)^{\frac{1}{2}}\right)}{2(1-f)^2\frac{2}{n^2\pi}cos(\alpha)}.$$

To calculate our overall absorption for *real world optimised*, G_a, G_b and G_c are evaluated at each incident solar flux energy in turn (using AM1 incident solar flux). The model which gives the largest value of G i.e., the maximum light absorbed at that incident energy, is then used, and the overall absorption model is obtained by stitching the solutions for all energies together. For *sun travel region* model the stronger light absorption from only option a) or b) is instead used. In general a) is the best solution when absorption is strong ($f \sim 1$), which transistions to solution b) and then c) as $f \to 0$. We note that, in model c), if $\theta_x < \frac{\pi}{2} - \alpha + \theta_{sun}$ then the model breaks down as the absorption angle is less than the angle the sun subtends in the sky (while we have modelled the sun as a point source). To account for this in simulations, we multiply the incident direct flux by $\frac{\theta_x - \frac{\pi}{2} + \alpha}{\theta_{\text{sun}}}$ in this case.

As an example of the *real world optimised* model, in Figure S4 we present the polar angles at which a directional filter should transmit light (for all azimuthal angles) as a function of energy for a 500 nm $MAPbI_3$ thin film. The maximum transmission angle can be seen to change rapidly at approximately 1.6 eV, corresponding to the change between solution a) and b). As the energy is further reduced the transmission region moves to two specific angular regions, while the transmission at $\theta = 90°$ goes to 0 – this is the transition between solution b) and c).

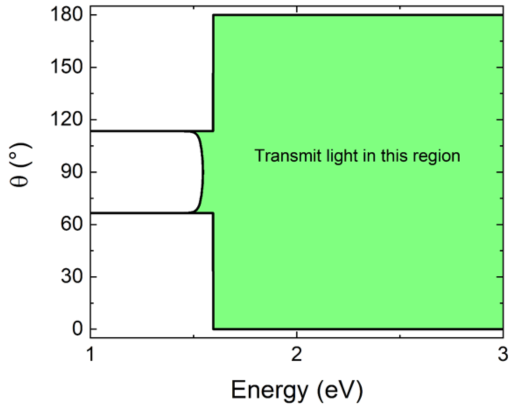

Figure S4. The polar angles at which a directional filter should transmit light for a 500 nm $MAPbI_3$ thin film. We note that when light is transmitted it is transmitted for all ϕ angles.

Supporting Information Note 4 – Silicon results

We present efficiency results for silicon solar cells and Supporting Table S1. We note that *Real world optimised* produces an extra 12.4 kWhm^{-2} throughout the year when compared to *Directionless*.

Absorptance model	AM1.5 efficiency (%)	Optimal AM1.5 thickness (µm, ±1 µm)	Equatorial efficiency over 1 year (%)	Optimal thickness (µm, ±2 µm)
Directionless	29.38	96	28.74	74
Sun only	32.40	20	26.83	> 490
Sun travel region	29.75	60	29.11	46
Real world optimised	29.62	56	29.14	46

Supporting Table S1. Equivalent of main text results for silicon, alongside optimal thickness for each structure.

We present absorptance, AM1.5 solar cell efficiency and real world equatorial efficiencies for silicon in Figures S5-S7.

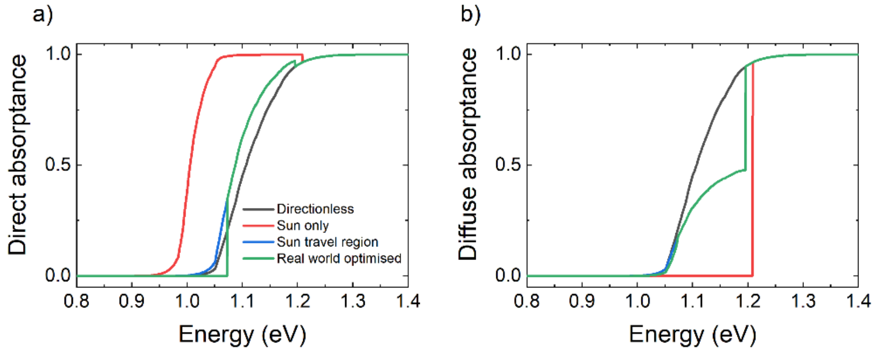

Figure S5. Direct and diffuse absorptances for a 100 µm silicon absorber, for the four absorptance models considered in the main text. Legend in a) applies to both plots.

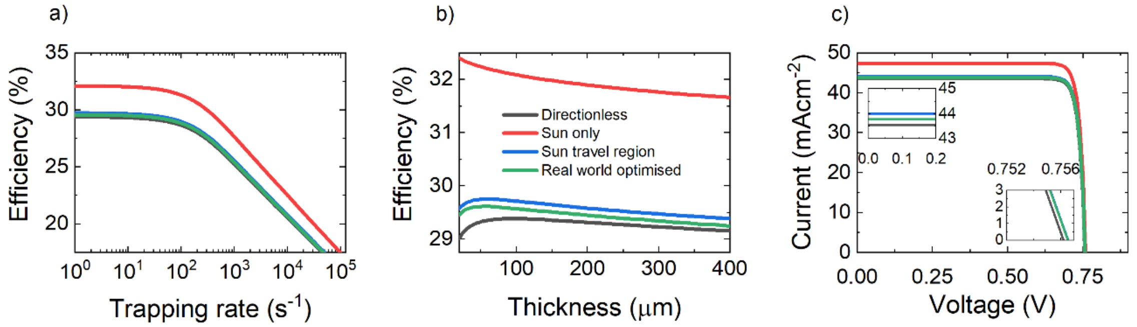

Figure S6. Efficiency with trapping rate, and with thickness for no trapping, for a 100 µm silicon absorber layer under AM1.5, is presented in a) and b) respectively. Current-voltage curves for no charge trapping and a 100 µm absorber layer are presented in c). Legend in b) applies to all Figures.

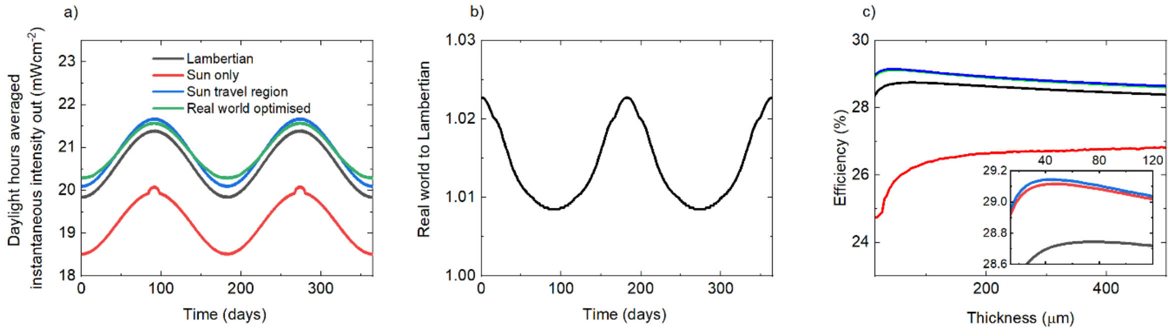

Figure S7. a) Daylight hours averaged instantaneous power out over the course of a year for the absorptance models considered for a 100 μm silicon absorber layer. b) presents the ratio of real world optimised to directionless. c) Efficiency throughout the year with thickness of the absorber layer. Inset shows optimal thickness region in more detail. Legend in a) applies to c).

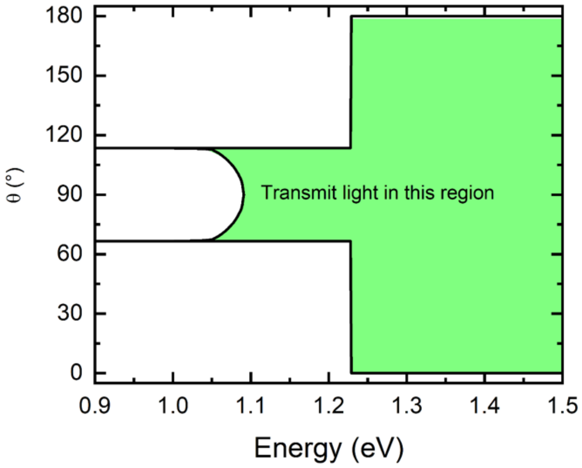

Figure S8. The polar angles at which a directional filter should transmit light for a 46 μm silicon absorber. We note that when light is transmitted it is transmitted for all ϕ angles.

Supporting Information Note 5 – AM1.5 results for $MAPbI_3$ in detail

To understand the potential of each absorptance model for solar panels under standard solar cell laboratory test conditions (AM1.5), we present the limiting efficiency of each $MAPbI_3$ cell as a function of thickness and charge trapping rate in Figures S9a and b respectively. It can be seen that the directionless model gives the lowest efficiency of 30.9 % at 500 nm thickness, while the sun only model, which is designed to be optimal under direct AM1.5-type illumination, has the highest efficiency of 35.3 % (see main text Table 1 for all values). All models respond very similarly to different absorber thicknesses or charge trapping rates (Figure S9a and b), meaning the benefits of controlling absorption direction is maintained for non-ideal solar cells. To better understand the improvements in solar cell performance, we plot current-voltage curves for all models in Figure S9c, for a 500-nm-thick film with no charge trapping. We find both short-circuit current and open-circuit voltage are enhanced by these absorptance models. The increase in short circuit current is expected, as these models are designed to absorb more light than directionless absorption. The open-circuit voltage (which is proportional to $\ln\left(\frac{J_{SC}}{J_0}\right)$) increases not only due to increased short circuit current density, but reduced recombination current densities as diffuse absorptance is reduced near the bandgap (main text Figure 2f).

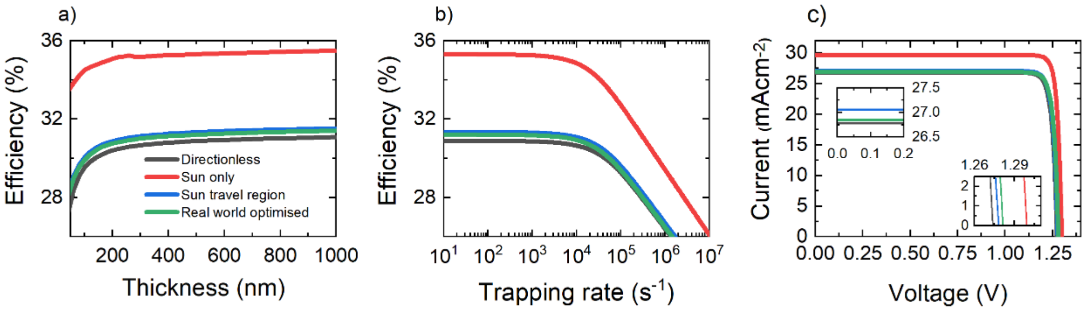

Figure S9. The limiting efficiency of a $MAPbI_3$ solar cell as a function of thickness, and trapping rate for a 500 nm film, are presented in a) and b) respectively. Current-voltage curves of each model are presented in c). Legend in a) applies to all plots.

Supporting Information Note 6 – discussion of spectrum and results with reduced direct irradiation

Directional filters will be most beneficial to a solar cell when a significant portion of the incident light comes from direct and circumsolar radiation. While it is beyond the scope of this work to explore the benefits of directional filters at many points on the earth's surface using local direct and diffuse irradiance, here we discuss the magnitude of efficiency improvements that can be anticipated when the portion of incident radiation that is direct is reduced. Specifically, we again model a 500 nm thick $MAPbI_3$ absorber layer but the direct component of incident AM1 is varied relative to that presented in Figure S3 while the diffuse component is kept constant. We present the change in annual efficiency, relative to a solar cell with no directional filter, for different AM1 direct components in Figure S10.

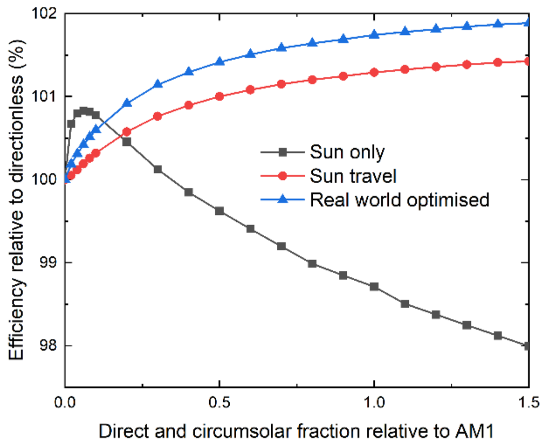

Figure S10. Efficiency of a 500 nm thick $MAPbI_3$ absorber layer with different directional filters relative to the efficiency of a solar cell with no directional filter, as the component of direct and circumsolar incident radiation is varied relative to AM1. Here a direct and circumsolar fraction of '1' corresponds to AM1 presented in Figure S3.

As the direct and circumsolar fraction of the incident radiation is reduced the benefit of the *sun travel* and *real world optimised* directional filters is reduced. However, in all cases there is still a benefit, and when the direct and circumsolar radiation is greater than 20 % of AM1 (which corresponds to cloudier conditions) there is still a relative efficiency increase of 1 %. Therefore, directional filters result in efficiency increases even when direct and circumsolar incident radiation is much lower than that presented in the models elsewhere in this work. Importantly, when there is no direct irradiance these models give no efficiency increase relative to the *directionless* model.

We also note that as the direct and circumsolar fraction of AM1 is reduced, the efficiency from the *Sun only* model increases and, for low direct fractions, surpasses that of the *directionless* model. This is caused by this filter creating a sharper absorption edge than the directionless model, effectively increasing the bandgap of the absorber to one better suited to the diffuse (i.e. much bluer) incident spectrum (see Figure 2f for the diffuse absorption edge). In other words this higher efficiency is due to a voltage effect, not a change in the number of absorbed photons. At extremely low direct irradiances the light absorbed when the sun is perpendicular to the panel is so low that the *Sun only* model becomes equivalent to the *directionless* model (see Supporting Information Note 3 for details on *Sun only* model), meaning this model gives no efficiency increase for zero direct irradiance.

Supporting Information Note 7 – Monte-Carlo ray tracing

We implemented ray tracing on curved glass surfaces (within periodic boundary conditions, to allow for surfaces that tesselate) to explore the possibility of using lenses to achieve the required directional functionality. As light can be incident at any point on the surface and from any incident angle (4 parameters) we used a Monte-Carlo approach to calculate the response of surfaces rapidly. Initially we tried to create a surface which directs light incident from the region $\theta = 90° - \alpha$ to $90° + \alpha$ into one spatial region and light from other angles into a different region at some plane behind the glass (i.e. no requirement for a tight focusing spot). Placing spectral filters in these two regions would achieve the *sun travel region* model. We also recorded the power transmission through the lens for each lens shape. When varying surface shape we found that any approach that achieved greater splitting of light from the two angular regions also reduced the total transmitted light (due to total internal reflection within the glass), as presented in Figure S11. Thus, we concluded that for simple optical structures (1 or 2 curved surfaces) it is not possible to segregate light from different angles into two regions and simultaneously maintain near-unity transmission.

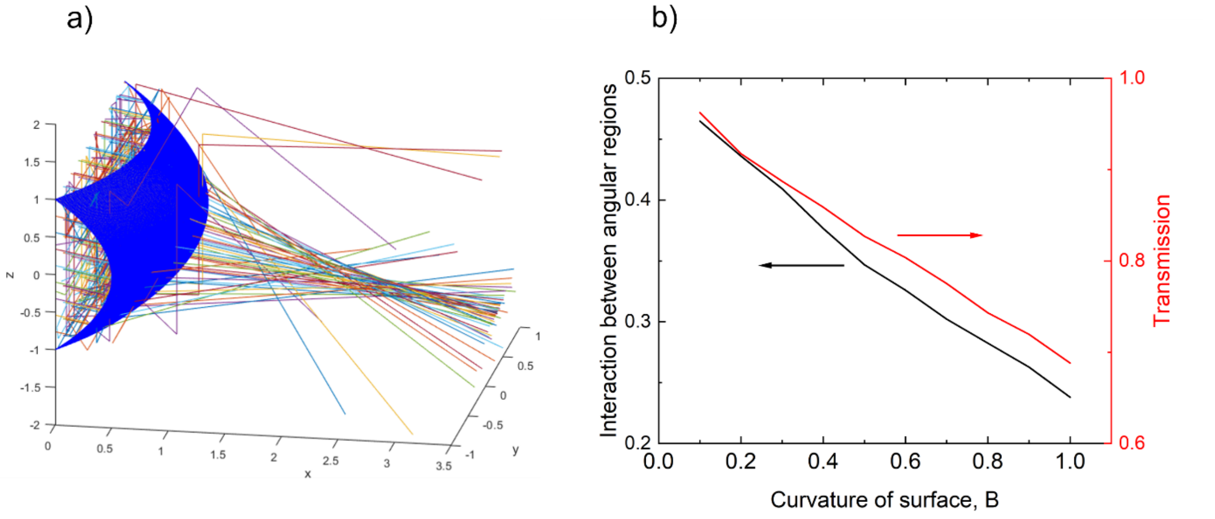

Figure S11. a) Example of ray tracing result for a curved surface (blue). b) The interaction between the two incident angular regions (defined as the fraction of total beams incident on the 'wrong' area of the focal plane), and the transmission, as a function of the parameter B describing the curvature of the glass surface (higher B is greater curvature). In this simulation the simulated surface is $x = 1 - \frac{1}{2}B(y^2 + z^2)$.

Supporting Information Note 8 – Luneburg lens transformation

The approach taken here follows the description of Schurig et al.[8] (noting other similar transforms have been discussed in several contexts[9,10]). We work in cylindrical polars and, for this Supporting Note only, we take the *z* direction to be that which light is incident from, as this makes the transformation simpler to write down.

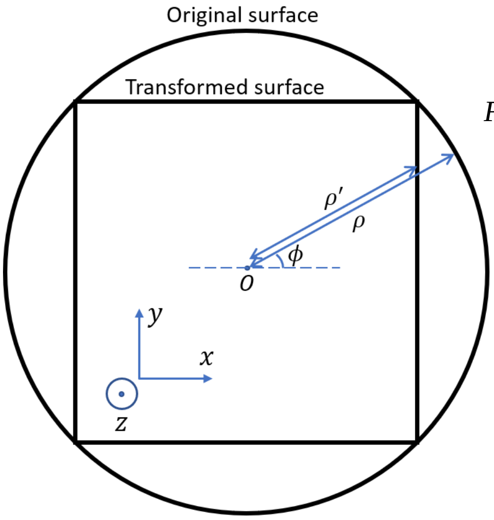

Figure S12. Top view schematic of the original and transformed surfaces.

We require a transformation that moves all regions outside the square into the square (see top view of problem in Figure S12). The z heights at which part of the sphere is outside the square (defining $z = 0$ to be the sphere centre) are given by

$$|z| = z_{\mathrm{lim}} = \begin{cases} R\left(1 - \frac{1}{2}\sec(\phi)^2\right)^{\frac{1}{2}} \; when \; |\cos(\phi)| \geq \frac{1}{\sqrt{2}} \\ R\left(1 - \frac{1}{2}\mathrm{cosec}(\phi)^2\right)^{\frac{1}{2}} \; otherwise \end{cases}$$

where R is the radius of the cylinder. Thus, we suggest the following transform in the region where $|z| < z_{\mathrm{lim}}$:

$$\rho' = \begin{cases} \frac{R\rho}{\sqrt{2(R^2 - z^2)}|\cos(\phi)|} \; for \; |\cos(\phi)| \geq \frac{1}{\sqrt{2}} (defined\ as\ j = 0) \\ \frac{R\rho}{\sqrt{2(R^2 - z^2)}|\sin(\phi)|} \; otherwise \; (defined\ as\ j = 1) \end{cases}$$

$$\phi' = \phi, z' = z.$$

To explain this transform we consider the case where $|\cos(\phi)| \geq \frac{1}{\sqrt{2}}$ and note that on the sphere boundary here $z = \sqrt{R^2 - \rho^2}$, meaning that in the transformed coordinate system on the boundary $\rho' = \frac{R}{\sqrt{2}|\cos(\phi)|}$. Therefore, within the transformed coordinates the boundary is given by $x' = \rho' \cos(\phi') = \frac{R}{\sqrt{2}}$ i.e. the side of a square with length $\frac{2R}{\sqrt{2}}$. Furthermore, we note that this transform does not affect the top surface of the sphere (the part which interacts with the surroundings). Within this transformation we can state

$$\frac{\partial \rho'}{\partial \rho} = \frac{\rho'}{\rho}$$

$$\frac{\partial \rho'}{\partial \phi} = (-1)^j \rho' \tan(\phi)^{-1^j}$$

$$\frac{\partial \rho'}{\partial z} = \frac{\rho' z}{R^2 - z^2}.$$

This allows us to write

$$\Lambda_i^{i'} = \begin{pmatrix} \frac{\rho'}{\rho} & (-1)^j \rho' \tan(\phi)^{-1^j} & \frac{\rho' z}{R^2 - z^2} \\ 0 & 1 & 0 \\ 0 & 0 & 1 \end{pmatrix}$$

and

$$\Lambda_{\hat{\imath}}^{\hat{\imath}'} = \begin{pmatrix} \frac{\rho'}{\rho} & \frac{\rho'}{\rho}(-1)^j \tan(\phi)^{-1^j} & \frac{\rho' z}{R^2 - z^2} \\ 0 & \frac{\rho'}{\rho} & 0 \\ 0 & 0 & 1 \end{pmatrix},$$

where $\Lambda_i^{i'} = \frac{\delta x^{i'}}{\delta x^i}$, where x^i is a coordinate in the coordinate basis, and $\hat{\imath}$ corresponds to a coordinate in the coordinate basis (see Schurig et al. for further discussion)[8]. Consequentially,

$$\det\left(\Lambda_{\hat{\imath}}^{\hat{\imath}'}\right)^{-1} \Lambda_{\hat{\imath}}^{\hat{\imath}'} \Lambda_{\hat{\jmath}}^{\hat{\jmath}'} = \begin{pmatrix} 1 + \tan(\phi)^{-1^j \times 2} + \frac{\rho^2 z^2}{(R^2 - z^2)^2} & (-1)^j \tan(\phi)^{-1^j} & \frac{\rho^2 z}{\rho'(R^2 - z^2)} \\ (-1)^j \tan(\phi)^{-1^j} & 1 & 0 \\ \frac{\rho^2 z}{\rho'(R^2 - z^2)} & 0 & \frac{\rho^2}{\rho'^2} \end{pmatrix}$$

and we note the electric permittivity in the transformed (primed) coordinates is $\varepsilon^{\hat{\imath}'\hat{\jmath}'} = \det\left(\Lambda_{\hat{\imath}}^{\hat{\imath}'}\right)^{-1} \Lambda_{\hat{\imath}}^{\hat{\imath}'} \Lambda_{\hat{\jmath}}^{\hat{\jmath}'} \varepsilon^{\hat{\imath}\hat{\jmath}}$. We also note that following Shurig et al.'s previous treatment of Luneburg lenses[8]

in transformed coordinates we assume $\varepsilon^{\hat{\iota}'\hat{\jmath}'} = \mu^{\hat{\iota}'\hat{\jmath}'} = n(\rho, z)\det\left(\Lambda_{\hat{\iota}}^{\hat{\iota}'}\right)^{-1}\Lambda_{\hat{\iota}}^{\hat{\iota}'}\Lambda_{\hat{\jmath}}^{\hat{\jmath}'}$, where μ is the magnetic permeability, and $n(\rho, z)$ is the refractive index as a function of position in the non-transformed coordinate basis. Finally, for a Luneburg lens in free space[8]

$$n(\rho, z) = \sqrt{2 - \frac{\rho^2 + z^2}{R^2}}.$$

We wrote a short code to calculate $\varepsilon^{\hat{\iota}'\hat{\jmath}'}$ at every position (as no analytical solution could be found) and then numerically diagonalized the matrix to find the local principle basis, which is what we plot in the main text. We note that in this transformed system there are some regions where $\varepsilon^{\hat{\iota}'\hat{\jmath}'} < 0.4$, implying a refractive index of less than 1. Thus, we suggest that any realisation of this filter would require it to be embedded in a higher index (> $\frac{1}{0.4}$) refractive index material, with filters shifted by appropriate amounts to account for this alternative surrounding material. Anti-reflection coatings could be used between this material and the surroundings, so this system could still be lossless.

References

(1) Green, M. A. Lambertian Light Trapping in Textured Solar Cells and Light-Emitting Diodes: Analytical Solutions. *Progress in Photovoltaics: Research and Applications* **2002**, *10* (4), 235–241. https://doi.org/10.1002/pip.404.

(2) National Renewable Energy Laboratory (NREL). *AM1.5*. https://www.nrel.gov/grid/solar-resource/spectra-am1.5.html (accessed 2020-06-29).

(3) Gueymard, C. A. Parameterized Transmittance Model for Direct Beam and Circumsolar Spectral Irradiance. *Solar Energy* **2001**, *71* (5), 325–346. https://doi.org/10.1016/S0038-092X(01)00054-8.

(4) Gueymard, C. A. SMARTS, A Simple Model of the Atmospheric Radiative Transfer of Sunshine: Algorithms and Performance Assessment., 1995.

(5) *SMARTS: Simple Model of the Atmospheric Radiative Transfer of Sunshine*. https://www.nrel.gov/grid/solar-resource/smarts.html (accessed 2023-11-11).

(6) Bowman, A. R.; Lang, F.; Chiang, Y. H.; Jiménez-Solano, A.; Frohna, K.; Eperon, G. E.; Ruggeri, E.; Abdi-Jalebi, M.; Anaya, M.; Lotsch, B. V.; Stranks, S. D. Relaxed Current Matching Requirements in Highly Luminescent Perovskite Tandem Solar Cells and Their Fundamental Efficiency Limits. *ACS Energy Letters* **2021**, *6*, 612–620. https://doi.org/10.1021/acsenergylett.0c02481.

(7) Yablonovitch, E. Statistical Ray Optics. *Journal of the Optical Society of America* **1982**, *72* (7), 899–907.

(8) Schurig, D. An Aberration-Free Lens with Zero F-Number. *New J. Phys.* **2008**, *10* (11), 115034. https://doi.org/10.1088/1367-2630/10/11/115034.

(9) Schurig, D.; Pendry, J. B.; Smith, D. R. Calculation of Material Properties and Ray Tracing in Transformation Media. *Opt. Express* **2006**, *14* (21), 9794–9804. https://doi.org/10.1364/OE.14.009794.

(10) Kundtz, N.; Smith, D. R. Extreme-Angle Broadband Metamaterial Lens. *Nature Mater* **2010**, *9* (2), 129–132. https://doi.org/10.1038/nmat2610.
